# Brentuximab Vedotin Retreatment in Patients with Relapsed or Refractory Classical Hodgkin Lymphoma or Peripheral T-Cell Lymphoma: A Retrospective United States Claims Analysis

**DOI:** 10.3390/curroncol31050195

**Published:** 2024-05-02

**Authors:** Dahlia Sano, Nicholas Liu, Scott Knowles, Joanna P. MacEwan, Shu Wang, Jenifer Wogen, Kristina S. Yu, Seung Tae Lee

**Affiliations:** 1 1500 East Medical Center Dr., University of Michigan, Ann Arbor, MI 48109, USA; 2 Pfizer Inc., 21823 30th Drive SE, Bothell, WA 98021, USA; 3 Genesis Research, 111 River St., Hoboken, NJ 07030, USA; 4 Greenebaum Comprehensive Cancer Center, University of Maryland School of Medicine, 22 South Greene St. S9D04A, Baltimore, MD 21201, USA

**Keywords:** cHL, CD30-expressing PTCL, real-world, claims database, treatment patterns

## Abstract

Brentuximab vedotin (BV) monotherapy (BV-M) and combination (BV-C) therapies are safe and effective for classical Hodgkin lymphoma (cHL) and CD30-expressing peripheral T-cell lymphomas (PTCLs). Although the sample sizes have been small (12–29 patients), in clinical studies, response rates of 53–88% have been reported for BV retreatment in patients with an initial BV response. We evaluated the real-world characteristics and treatment patterns of cHL/PTCL patients who received BV and were retreated in the United States. Symphony Health Patient Claims (11/2013–1/2022) were retrospectively analyzed to identify cHL/PTCL patients treated with BV and retreated with BV-M, BV-C, or non-BV therapy. Patient characteristics were described by retreatment, and predictors of BV-M retreatment were identified. Among the cHL and PTCL patients treated with BV (*n* = 6442 and 2472, respectively), 13% and 12%, respectively, were retreated with BV; the median times from initial BV to BV-M retreatment were 5 and 7 months, respectively; and the numbers of BV-M retreatment doses were 4 and 5, respectively. Among cHL patients, the predictors of BV-M retreatment were age (18–39 vs. ≥60 years), sex (women vs. men), and previous stem cell transplantation (yes vs. no). Among PTCL patients, the only predictor of BV-M retreatment was systemic anaplastic large-cell lymphoma subtype (yes vs. no). Real-world data support clinical study results suggesting earlier BV treatment be considered, as BV retreatment may be an option.

## 1. Introduction

In the United States, brentuximab vedotin (BV) in combination with chemotherapy is approved for adults with previously untreated stage III/IV classical Hodgkin lymphoma (cHL) and for adults with previously untreated CD30-expressing peripheral T-cell lymphoma (PTCL) [1]. As a monotherapy, BV (BV-M) is approved for adults with cHL or CD30-expressing PTCL who have failed prior multiagent chemotherapy regimens [1].

In ECHELON-1, adults with previously untreated stage III/IV cHL randomized to A + AVD (BV with doxorubicin, vinblastine, and dacarbazine) versus ABVD (doxorubicin, bleomycin, vinblastine, and dacarbazine) demonstrated a 41% reduction in the risk of death after a median follow-up of 73 months (6-year overall survival (OS), 93.9% vs. 89.4%; hazard ratio (HR) for death, 0.59; 95% CI, 0.40–0.88; *p* = 0.009) and a 32% reduction in the risk of disease progression or death (progression-free survival (PFS), 82.3% vs. 74.5%; HR, 0.68; 95% CI, 0.53–0.86) [2]. In ECHELON-2, adults with previously untreated CD30-expressing PTCL randomized to A + CHP (BV with cyclophosphamide, doxorubicin, and prednisone) versus CHOP (cyclophosphamide, doxorubicin, vincristine, and prednisone) demonstrated clinically meaningful improvements in 5-year OS (70.1% vs. 61.0%; HR, 0.72; 95% CI, 0.53–0.99) and 5-year PFS (51.4% vs. 43.0%; HR, 0.70; 95% CI, 0.53–0.91) [3]. Positive responses were also documented with BV-M in adults with previously treated cHL (overall response rate (ORR), 75%) [4] or systemic anaplastic large-cell lymphoma (sALCL), an aggressive PTCL subtype that universally expresses CD30 (ORR, 86%) [5].

Following an initial response to BV therapy, BV retreatment may be an option, with ORRs of 53–60% (complete response (CR) rate, 18–30%) in cHL [6,7,8] and 59–88% (CR rate, 38–67%) in CD30-expressing PTCL [3,6,7,8], with higher ORRs (63–100%) and CR rates (42–75%) reported for patients with sALCL [3,6,7,8].

As sample sizes have been small in clinical studies (12–29 patients) [3,6,7,8] and real-world BV-retreatment data are sparse, we evaluated the real-world characteristics and treatment patterns of patients with cHL or PTCL treated with a BV regimen and retreated with BV-M, BV combination therapy (BV-C), or non-BV therapy in the United States. Predictors of BV-M retreatment in patients with cHL or PTCL were also identified.

## 2. Materials and Methods

### 2.1. Study Design

**Figure 1:**
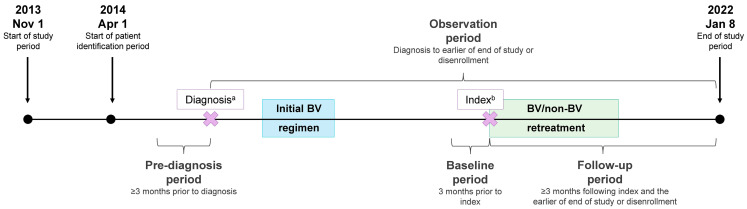
Study design. ^a^, the diagnosis date was defined as the date of the first cHL or PTCL claim during the patient identification period; ^b^, the index date was defined as the start date for retreatment with BV-M, BV-C, or non-BV therapy (i.e., index treatment).

Patient medical and pharmacy claims within the Symphony Health database were retrospectively analyzed to identify patients with cHL or PTCL initially treated with a BV regimen and retreated with BV-M, BV-C, or non-BV therapy (Figure 1). The Symphony Health database is a geographically representative database that captures a substantial portion of total medical activity in the United States. The database consists of claims submitted by physicians and pharmacies in the United States for reimbursement of services rendered to patients using CMS1450 and CMS1500 forms for medical claims and NCPDP forms for pharmacy claims. All payment types (cash, commercial, Medicare, Medicaid, and assistance programs) and all plans are represented. Patients are uniquely identified and can be tracked over 10 years across all practice settings. More than 95% of claims are linked to a unique practitioner.

During the study period (1 November 2013–8 January 2022), patients with a first cHL or PTCL claim (diagnosis date) starting on 1 April 2014 were identified to allow for the evaluation of ≥3 months of data prior to first cHL or PTCL diagnosis (i.e., pre-diagnosis period with no cHL or PTCL claims). Following the first cHL or PTCL diagnosis, patients treated with an initial BV regimen and then retreated with BV-M, BV-C, or non-BV therapy were identified. The index date was the start date for retreatment with BV-M, BV-C, or non-BV therapy (i.e., index treatment). The study baseline period was the 3-month period preceding the index date, and the follow-up period was at least 3 months following the index date to the earlier of the end of the study or disenrollment.

As this was a non-interventional, retrospective claims database analysis, informed consent or review and approval by an ethics committee were not required. The data evaluated were deidentified and could not be traced back to individual patients.

### 2.2. Study Population

Patients had a cHL or PTCL diagnosis identified by ICD-9/10 codes with ≥1 inpatient claim or ≥2 outpatient claims separated by ≥1 day and were at least 18 years of age at index. Additionally, patients were treated with an initial BV regimen and retreated with BV-M, BV-C, or non-BV therapy identified by either a treatment gap of ≥90 days or a change in treatment. Patients needed ≥3 months of continuous health plan enrollment prior to the cHL or PTCL diagnosis date and ≥3 months of continuous health plan enrollment following the index date.

Patients with ≥1 BV claim during the 3-month pre-diagnosis period were excluded from the analysis. For patients with cHL, those whose initial and index treatments were A + AVD or index treatment was A + CHP were excluded. For patients with PTCL, those whose initial and index treatments were A + CHP were excluded.

### 2.3. Outcomes

The proportions of patients receiving an initial BV regimen and retreatment with BV-M, BV-C, or non-BV therapy were calculated. The demographics included age, sex, and treatment region, and clinical characteristics included index year, diagnosis year, and previous treatment history including stem cell transplantation (SCT), PTCL subtypes, and Quan-enhanced Charlson Comorbidity Index score. Time from index date to the end of follow-up was captured. Treatment regimens were identified by treatment procedure and National Drug Code and categorized as BV or non-BV.

### 2.4. Statistical Analysis

Patient demographics, clinical characteristics, treatment regimens, and treatment profiles were described descriptively and stratified by diagnosis. Summary statistics were calculated, including frequencies and percentages for categorical variables and medians and interquartile ranges for continuous variables.

Logistic regression was performed to estimate the odds ratios (ORs) and 95% confidence intervals for the patient characteristics associated with BV-M versus BV-C or non-BV retreatment. The regression models included the following independent variables: age, sex, and receipt of SCT prior to index. PTCL subtype was also included in the PTCL model.

## 3. Results

### 3.1. cHL Cohort

**Figure 2:**
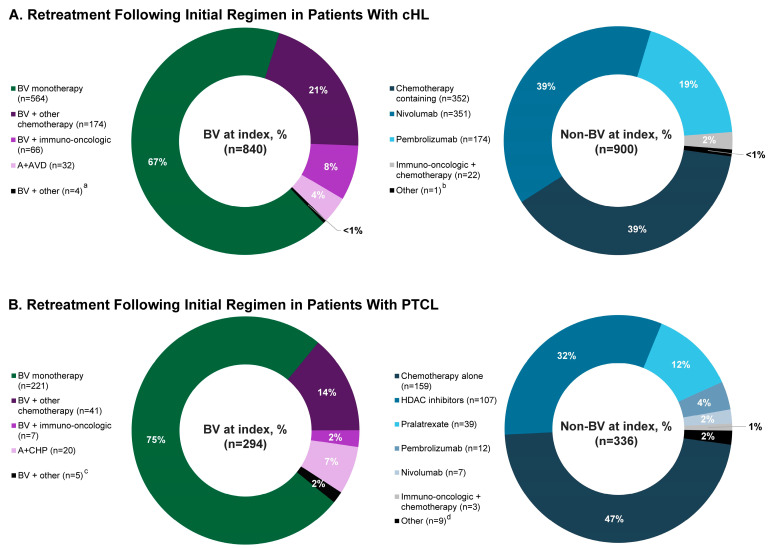
Retreatment at index in patients with (**A**) cHL or (**B**) PTCL treated with an initial BV regimen. Because of rounding, percentages may not total 100. ^a^, bendamustine, brentuximab vedotin, and pembrolizumab, *n* = 1; brentuximab vedotin, dacarbazine, doxorubicin, nivolumab, and vinblastine, *n* = 1; brentuximab vedotin, dacarbazine, doxorubicin, pembrolizumab, and vinblastine, *n* = 1; brentuximab vedotin, doxorubicin, gemcitabine, nivolumab, and vinorelbine, *n* = 1. ^b^, nivolumab and pembrolizumab, *n* = 1. ^c^, brentuximab vedotin and belinostat, *n* = 1; brentuximab vedotin, bendamustine, and pembrolizumab, *n* = 1; brentuximab vedotin and romidepsin, *n* = 3. ^d^, bortezomib, *n* = 4; lenalidomide, *n* = 5. **Abbreviations**: A + AVD, brentuximab vedotin in combination with doxorubicin, vinblastine, and dacarbazine; A + CHP, brentuximab vedotin in combination with cyclophosphamide, doxorubicin, and prednisone; BV, brentuximab vedotin; cHL, classical Hodgkin lymphoma; HDAC, histone deacetylase inhibitor–based therapy; PTCL, peripheral T-cell lymphoma.

Among patients with cHL treated with an initial BV regimen (N = 6442) and then retreated, 13% (*n* = 840) received BV at index and were included in either the BV-M (9% (*n* = 564)) or BV-C (4% (*n* = 276)) group; 14% (*n* = 900) received a non-BV therapy at index and were included in the non-BV group (Figure 2A).

#### 3.1.1. BV-M Retreatment at Index

The median age at diagnosis was 39 years and 52% of patients were men (Table 1). The median time from initial BV regimen to BV-M retreatment was 5 months. The initial BV regimen was A + AVD for 5% of patients and 12% of patients received SCT pre-index. The median (IQR) time from BV-M retreatment at index to the end of follow-up was 31 (15–47) months. During retreatment, the median (IQR) number of BV-M doses was 4 (2–9), and 81% of BV-M retreatment at index occurred in the second- and third-line settings (Table 2).

Of the patients in the BV-M retreatment group, 31% subsequently received another systemic therapy (BV-M, 10%; BV-C, 1%; non-BV, 19%; Table 3), with a median time to subsequent therapy of 7 months. No patients received autologous SCT and 0.5% received allogeneic SCT (Table 3). The median time to subsequent SCT was 5 months.

#### 3.1.2. BV-C Retreatment at Index

The median age was 42 years and 54% of patients were men (Table 1). The median time from initial BV regimen to BV-C retreatment was 2 months. The initial BV regimen was A + AVD for 18% of patients and 4% of patients received SCT pre-index. The median (IQR) time from the start of BV-C retreatment at index to the end of follow-up was 24 (12–36) months, and the median number of BV doses was 4 (2–6). The therapies received by >10% of the BV-C group at index included BV + other chemotherapy (63%), BV +immuno-oncologic (24%), and A + AVD (12%).

Among the BV-C group, 39% subsequently received another systemic therapy (BV-M, 5%; BV-C, 11%; non-BV therapy, 23%; Table 3). The median time to subsequent systemic therapy was 6 months. The median time to subsequent SCT was 3 months; 2.5% of patients in the BV-C group subsequently received SCT, including 1.1% who received autologous SCT and 1.4% who received allogeneic SCT.

Among the 32 patients who received A + AVD at index, all had originally received BV frontline (BV-M, *n* = 18; BV + other chemotherapy, *n* = 13; and BV + immuno-oncologic, *n* = 1), with 30 receiving A + AVD in the second-line setting and 2 in the third-line setting. The median (IQR) time between frontline BV and A + AVD at index was 1 (0–1) month. Seven patients receiving A + AVD at index subsequently received systemic therapy (BV-M, *n* = 1; non-BV, *n* = 6 (chemotherapy-containing regimens, *n* = 2 and pembrolizumab monotherapy, *n* = 4)).

#### 3.1.3. Non-BV Retreatment at Index

The median age was 52 years and 61% of patients were men (Table 1). The median time from initial BV regimen to non-BV retreatment was 2 months. The initial BV regimen was A + AVD for 17% of patients; 5% of patients received SCT pre-index. The median (IQR) time from the start of non-BV retreatment at index to the end of follow-up was 25 (12–43) months. Index non-BV treatments received by >10% of the patients were chemotherapy alone (39%), nivolumab alone (39%), and pembrolizumab alone (19%).

Another systemic therapy was subsequently administered to 33% of patients in the non-BV group (Table 3). The median time to subsequent systemic therapy was 6 months. A subsequent SCT was received by 6.0% of patients in the non-BV retreatment group, including 4.4% who received autologous SCT and 1.6% who received allogeneic SCT. The median time to subsequent SCT was 3 months.

#### 3.1.4. Predictors of Retreatment with BV-M in cHL

**Figure 3:**
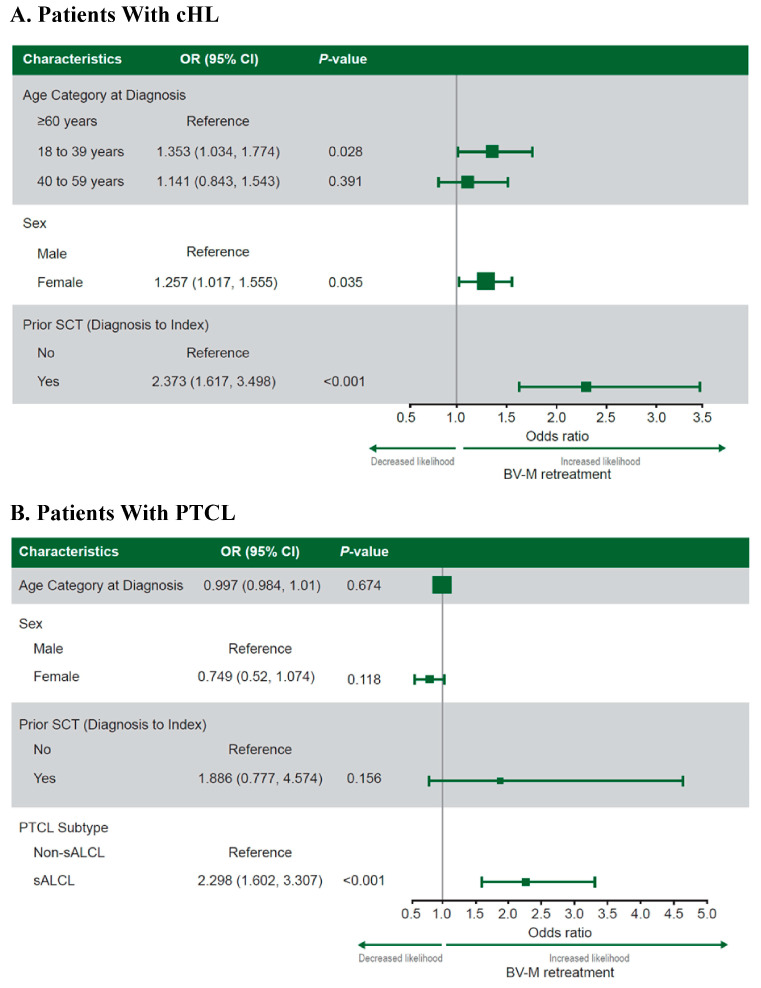
Predictors of BV-M versus BV-C or non-BV retreatment at index in patients with cHL (**A**) or PTCL (**B**) treated with an initial BV regimen. **Note:** Values of >1 indicate a greater likelihood of retreatment with BV-M than BV-C or non-BV therapy. The categorical variables included geographic region, year of initial BV regimen, and baseline individual Charlson Comorbidity Index score. The continuous variables included initial BV regimen line of therapy, follow-up time from diagnosis (cHL only), and age at diagnosis (PTCL only). **Abbreviations:** BV, brentuximab vedotin; BV-C, brentuximab vedotin combination therapy; BV-M, brentuximab vedotin monotherapy; cHL, classical Hodgkin lymphoma; OR, odds ratio; PTCL, peripheral T-cell lymphoma; sALCL, systemic anaplastic large-cell lymphoma; SCT, stem cell transplantation.

The predictors of BV-M versus BV-C or non-BV retreatment were age (18–39 vs. ≥60 years; OR, 1.353; 95% CI, 1.034–1.774; *p* = 0.028), sex (women vs. men: OR, 1.257; 95% CI, 1.017–1.555; *p* = 0.035), and previous SCT (SCT vs. no SCT pre-index: OR, 2.373; 95% CI, 1.617–3.498; *p* < 0.001; Figure 3A).

### 3.2. PTCL Cohort

Among patients with PTCL treated with an initial BV regimen (N = 2472) and then retreated, 12% (*n* = 294) received BV at index and were included in either the BV-M (9% (*n* = 221)) or BV-C (3% (*n* = 73)) group; 14% (*n* = 336) received a non-BV therapy at index and were included in the non-BV group (Figure 2B).

#### 3.2.1. BV-M Retreatment at Index

The median age at diagnosis was 62 years, 64% of patients were men, and 49% had a non-sALCL subtype (Table 1). The median time from initial BV regimen to index BV-M retreatment was 7 months. Eighteen percent of patients received A + CHP as their initial BV regimen and 5% of patients received SCT pre-index. The median (IQR) time from BV-M retreatment at index to the end of follow-up was 20 (10–32) months. During retreatment, the median (IQR) number of BV-M doses was 5 (2–8), and 88% of BV-M retreatment at index occurred in the second- and third-line settings (Table 2).

Another systemic therapy was subsequently administered to 40% of patients in the BV-M group (BV-M, 18%; BV-C, 1%; non-BV, 21%; Table 3), with a median time to subsequent therapy of 8 months. The median time to subsequent SCT was 4 months; 2.7% of patients subsequently received SCT, including 0.9% who received autologous SCT and 1.8% who received allogeneic SCT.

#### 3.2.2. BV-C Retreatment at Index

The median age was 57 years, 63% of patients were men, and 58% had a non-sALCL subtype (Table 1). The median time from initial BV regimen to BV-C retreatment was 1 month. Twenty-three percent of patients received A + CHP as their initial BV regimen. No patient received SCT pre-index. The median (IQR) time from start of index BV-C retreatment to the end of follow-up was 18 (11–26) months, with patients receiving a median (IQR) of 3 (2–5) BV doses. The most commonly administered index BV-C treatments were BV + other chemotherapy (56%), A + CHP (27%), and BV + immuno-oncologic (10%).

Among the BV-C group at index, 55% subsequently received another systemic therapy (BV-M, 6%; BV-C, 12%; non-BV, 37%; Table 3). The median (IQR) time to subsequent systemic therapy was 7 months. No patients subsequently received autologous SCT whereas 1.4% subsequently received an allogeneic SCT. The time to subsequent SCT was 5 months.

Among the 20 patients who received A + CHP at index, 15 had originally received BV frontline and 5 had received it as second-line therapy. The initial BV regimen was BV-M for 13 patients and BV + other chemotherapy for 7 patients. At index, 14 patients received A + CHP as second-line therapy and 6 patients received it as third-line therapy. The median (IQR) time between the initial BV therapy and index A + CHP was 1 (1–2) month. Seven patients who received index A + CHP subsequently received systemic therapy (BV-C, *n* = 1; non-BV therapy, *n* = 6 (chemotherapy-containing regimens, *n* = 3; romidepsin monotherapy, *n* = 2; and romidepsin-based therapy, *n* = 1)).

#### 3.2.3. Non-BV Retreatment at Index

The median age was 64 years, 57% of patients were men, and 73% had a non-sALCL subtype (Table 1). The median time was 3 months from initial BV regimen to non-BV retreatment. The initial BV regimen for 24% of patients was A + CHP. Pre-index, 4% of patients received SCT. The median (IQR) time from start of non-BV retreatment at index to the end of follow-up was 12 (7–23) months. Non-BV treatments received at index by >10% of patients were chemotherapy alone (47%), histone deacetylase inhibitors (32%), and pralatrexate (12%).

Forty percent of patients in the non-BV therapy group at index subsequently received another systemic therapy (Table 3). The median time to subsequent systemic therapy was 4 months. The median time to subsequent SCT was 3 months; 2.7% of the patients subsequently received SCT, including 1.2% who received autologous SCT and 1.5% who received allogeneic SCT.

#### 3.2.4. Predictors of Retreatment with BV-M in PTCL

The only predictor of BV-M versus BV-C or non-BV retreatment identified was PTCL subtype (sALCL vs. non-sALCL subtypes: OR, 2.298; 95% CI, 1.602–3.307; *p* < 0.001; Figure 3B).

## 4. Discussion

The results from this real-world analysis show that BV retreatment may be an option for some patients with cHL or PTCL. The safety and efficacy of frontline BV in combination with chemotherapy for adults with newly diagnosed stage III/IV cHL or previously untreated CD30-expressing PTCLs was established in the ECHELON-1 and ECHELON-2 trials. For patients with cHL who progress following frontline therapy, treatment options are limited and based on SCT eligibility. For patients with PTCL who progress following frontline therapy, treatment options are lacking. Clinical trial data suggest that BV retreatment may be an option following a response (typically >6 months) to an initial BV regimen in cHL (ORR, 53–60%; CR rate, 18–30%) [6,7,8] and CD30-expressing PTCLs (ORR, 59–88%; CR rate, 38–67%) [3,6,7,8].

In ECHELON-1 (A + AVD, *n* = 662) [2], 10 patients initially responding to A + AVD were retreated with BV (BV-M, *n* = 8; BV-C, *n* = 2) following disease progression, but neither treatment duration nor ORR were reported. In ECHELON-2 (A + CHP, *n* = 226) [3], 29 patients initially responding to A + CHP were retreated with ≥1 BV regimen following disease progression (BV-M, *n* = 25; BV-C, *n* = 4). The median time from frontline therapy to BV retreatment was 15 months, the ORR was 59% (CR rate, 38%), and the median treatment duration was 2.1 months.

BV retreatment was evaluated in several small clinical studies. An open-label, multicenter, international, phase 2 study evaluated BV retreatment following ≥2 systemic therapies (median of 4), including an initial BV regimen [6]. Patients retreated with BV received a median of 7 BV cycles. Among patients with Hodgkin lymphoma (*n* = 21; response assessment, *n* = 20) or sALCL (*n* = 8), the median time between the initial and subsequent BV regimens was 11.4 and 4.7 months, respectively; the ORR was 60% (CR rate, 30%) and 88% (CR rate, 63%), respectively; the duration of response was 9.2 and 12.3 months, respectively; and the median PFS was 9.9 and 12.9 months, respectively. Median OS was not reached in either the Hodgkin lymphoma group or the sALCL group.

A multicenter, single-arm, phase 2 clinical study evaluated BV retreatment in adults with relapsed/refractory cHL or CD30-expressing PTCL who received an initial BV regimen ≥ 6 months pre-study enrollment and had an objective response followed by disease progression/relapse [8]. Eleven patients received ≥1 study drug dose, with a median of 8 BV retreatment doses administered over 25 weeks. Of the patients enrolled, 55% were Black/African American. Patients with cHL (*n* = 5) or PTCL (*n* = 6 (sALCL, *n* = 4)) retreated with BV had a median age of 31 (range, 28–55) and 63 (35–77) years, respectively; a median time from initial to subsequent BV therapy of 28.2 and 20.1 months, respectively; and an ORR of 60% (CR rate, 20%) and 83% (CR rate, 67%; sALCL: ORR, 100%; CR rate, 75%), respectively.

A retrospective, multicenter, observational study assessed the safety and efficacy of BV retreatment in cHL or sALCL patients from October 2014–April 2018 in Japan [7]. Among all patients, 6 BV doses were administered over 5.9 months. Patients with cHL (*n* = 18; efficacy assessment, *n* = 17) or sALCL (*n* = 10) achieving a CR or partial response before discontinuing their initial BV regimen received BV retreatment. Among the cHL and sALCL groups (median age, 44.0 and 66.5 years, respectively), the ORR was 52.9% (CR rate, 17.6%) and 70.0% (CR rate, 60.0%), respectively; the duration of response was 21.5 months and not reached, respectively; the median time to treatment failure was 5.3 and 6.1 months, respectively; and the PFS was 5.3 months and not reached, respectively. At data cutoff, 22% of cHL patients and 50% of sALCL patients were still receiving BV retreatment. Following BV retreatment in the cHL and sALCL groups, 9 and 2 patients, respectively, subsequently received another systemic therapy and 2 and 1 patients, respectively, subsequently received SCT.

To our knowledge, our real-world analysis is the largest to date in the United States to evaluate BV retreatment and includes data for 840 patients with cHL and 294 patients with PTCL retreated with BV. The patients with cHL retreated with BV were similar to those participating in other cHL BV retreatment trials, with most patients receiving BV-M as their initial BV therapy. This finding likely reflected treatment practices during our study period, which started in November 2013. The initial BV regimen for 5% of patients in the BV-M group and 18% of patients in the BV-C group was A + AVD, a preferred frontline regimen for stage III/IV cHL that received Food and Drug Administration approval in 2018 [9].

A greater proportion of patients with cHL received SCT prior to retreatment with BV-M compared to those who received retreatment with BV-C or non-BV therapies. We found that patients with a previous SCT were more likely to be retreated with BV-M than with BV-C or non-BV therapy, perhaps reflecting the use of BV maintenance therapy post-autologous SCT, with such use supported by the AETHERA trial results [10].

Among patients with cHL retreated with BV-M or BV-C in our analysis, 31% and 39%, respectively, received subsequent systemic therapy, and 0.5% and 2.5%, respectively, received subsequent SCT. Although these numbers were lower than the 50% and 11.1% of patients who received subsequent systemic therapy or subsequent SCT, respectively, in the retrospective study from Japan [7], we did find that approximately 11% of the BV-M group and 16% of the BV-C group received a subsequent round of BV as a third or later line of therapy.

The patients with PTCL in our analysis also had similar characteristics to clinical trial participants. We found that 51% of patients retreated with BV-M and 42% of patients retreated with BV-C had sALCL and that patients with versus without an sALCL subtype were more likely to be retreated with BV-M than with BV-C or non-BV therapy. This result was not surprising, as sALCLs universally express CD30 and BV is indicated in the relapsed/refractory setting for sALCL. However, BV-M is also guideline-recommended for CD30-expressing non-sALCL subtypes [11], such as PTCL-not otherwise specified (NOS; CD30 expression, ~60%) and angioimmunoblastic T-cell lymphoma (CD30 expression, ~50%) [12]. We found that 49% of participants treated with BV-M had a non-sALCL subtype, potentially reflecting a need for additional therapies for relapsed/refractory PTCL. Some of these patients potentially had CD30-expressing disease, as CD30 expression is not captured in the Symphony Health database.

As with the patients with cHL, most patients with PTCL in the BV-M and BV-C groups previously received BV-M, which was approved for sALCL in 2011. However, 18% and 23% of the BV-M and BV-C groups, respectively, initially received A + CHP, which was approved in 2018 for the frontline treatment of CD30-expressing PTCLs. In the BV-M and BV-C groups, 40% and 55%, respectively, subsequently received another systemic therapy, and 2.7% and 1.4%, respectively, received subsequent SCT. Among those treated with an initial BV regimen, approximately 19% in the BV-M group and 18% in the BV-C group subsequently received BV as a third or later line of therapy.

Finally, across the identified clinical and real-world studies, patients retreated with BV in the clinical studies received more BV retreatment doses than those in the real-world studies, a finding that was not unexpected. In the clinical studies by Bartlett [6] and Sano [8], patients received a median of 7–8 BV retreatment doses. In the real-world study by Fukuhara [7], patients received 6 BV retreatment doses, and, in our analysis, the patients received a median of 3–5 BV retreatment doses. This variance may merely reflect differences between real-world practice and clinical trial settings.

This study’s limitations include those intrinsic to retrospective claims analyses and include selection bias, reliance on complete and accurate coding, the impact of unmeasured characteristics (e.g., disease stage, biomarkers, clinical outcomes), and a lack of information on lines of therapy. Some patients could have received lead-in BV-M prior to receiving BV-C at index, as the median time from initial to index BV was 2 months for cHL and 1 month for PTCL. However, after excluding the small number of patients with cHL who received A + AVD at index and the small number of patients with PTCL who received A + CHP at index, which may represent a change in initial therapy versus a new line of therapy, our results continue to support BV retreatment for some patients.

Although the Symphony Health data represent a substantial proportion of all U.S. medical claims and provide important information on real-world characteristics and treatment utilization, the results may not be generalizable to all patients with cHL or PTCL or to all practice settings. For patients with PTCL, CD30 testing results are not available in claims data; however, most patients with PTCL retreated with BV in this study had a subtype (i.e., sALCL or PTCL-NOS) with a CD30 expression rate of ≥60% [12]. Additionally, the database does not include information on laboratory test results or clinical outcomes that include response rates and survival data. Lastly, treatment selection may have been influenced by formulary restrictions for BV and other treatment regimens and the standard of care during the study period (2013–2022). Further retrospective research is needed to understand real-world clinical outcomes in patients with cHL or PTCL.

## 5. Conclusions

In this retrospective administrative claims analysis, we found that patients with cHL or PTCL initially treated with a BV-containing regimen were retreated with BV. Patients with cHL retreated with BV-M received a median of 4 doses and those with PTCL received a median of 5 doses. Our real-world findings, when viewed in conjunction with those from clinical studies that have reported ORRs of 53–60% in patients with cHL and 59–88% in patients with CD30-expressing PTCL retreated with BV, suggest that BV retreatment may be a viable option for some patients, including those intolerant to aggressive therapy.

## Figures and Tables

**Table 1:** Baseline characteristics for patients with cHL or PTCL who received BV-M, BV-C, or non-BV retreatment at index.

	cHL (N = 6442)			PTCL (N = 2472)		
	BV-M *n* = 564	BV-C *n* = 276	Non-BV *n* = 900	BV-M *n* = 221	BV-C *n* = 73	Non-BV *n* = 336
Age at diagnosis, median (IQR), yr	39 (28–60)	42 (28–68)	52 (32–69)	62 (51–72)	57 (47–66)	64 (55–72)
Age range, *n* (%)						
18–39 years	285 (51)	127 (46)	337 (37)			
40–59 years	130 (23)	68 (25)	214 (24)			
≥60 years	149 (26)	81 (29)	349 (39)			
Male sex, *n* (%)	295 (52)	149 (54)	547 (61)	141 (64)	46 (63)	191 (57)
PTCL subtype, *n* (%)						
sALCL				112 (51)	31 (42)	92 (27)
Non-sALCL				109 (49)	42 (58)	244 (73)
PTCL-NOS				85 (38)	30 (41)	153 (46)
ATLL				18 (8)	7 (10)	71 (21)
Mature T/NK-cell lymphoma				6 (3)	3 (4)	20 (6)
Enteropathy-associated T-cell lymphoma				0 (0)	2 (3)	0 (0)
Initial BV regimen, *n* (%)						
A + AVD	29 (5)	49 (18)	155 (17)			
A + CHP				39 (18)	17 (23)	81 (24)
BV-M	380 (67)	140 (51)	632 (70)	164 (74)	39 (53)	222 (66)
BV + other chemotherapy ^a^	138 (24)	67 (24)	93 (10)	16 (7)	17 (23)	27 (8)
BV + immuno-oncologic	17 (3)	20 (7)	19 (2)	0 (0)	0 (0)	1 (<1)
BV + other	0 (0)	0 (0)	1 (<1)	2 (1)	0 (0)	5 (2)
Prior SCT, *n* (%)	68 (12)	11 (4)	46 (5)	12 (5)	0 (0)	13 (4)
Prior lines of therapy, median (IQR) ^b^	2 (1–2)	2 (1–2)	1 (1–2)	1 (1–2)	1 (1–2)	1 (1–2)
Time from initial BV regimen to index, mo						
Mean (SD)	8 (8)	7 (12)	6 (9)	11 (10)	3 (6)	5 (7)
Median (IQR)	5 (4–8)	2 (1–7)	2 (1–7)	7 (4–14)	1 (1–2)	3 (1–7)

Because of rounding, percentages may not total 100. ^a^, chemotherapies other than AVD in patients with cHL and CHP in patients with PTCL. ^b^, lines of therapy received prior to index date. **Abbreviations**: A + AVD, brentuximab vedotin in combination with doxorubicin, vinblastine, and dacarbazine; A + CHP, brentuximab vedotin in combination with cyclophosphamide, doxorubicin, prednisone; ATLL, adult T-cell leukemia lymphoma; BV, brentuximab vedotin; BV-C, brentuximab vedotin combination therapy; BV-M, brentuximab vedotin monotherapy; cHL, classical Hodgkin lymphoma; IQR, interquartile range; mo, months; NOS, not otherwise specified; PTCL, peripheral T-cell lymphoma; sALCL, systemic anaplastic large-cell lymphoma; SCT, stem cell transplantation; SD, standard deviation; yr, years.

**Table 2:** BV-M retreatment at index by line of therapy in patients with cHL or PTCL.

BV-M Retreatment at Index	Initial BV Regimen for cHL, *n* (%)				
	Overall N = 564	1L *n* = 278	2L *n* = 219	3L *n* = 58	4L *n* = 9
2L	195 (35)	195 (70)	-	-	-
3L	262 (46)	74 (27)	188 (86)	-	-
4L	79 (14)	6 (2)	28 (13)	45 (78)	-
5L	18 (3)	3 (1)	2 (1)	8 (14)	5 (56)
6L	7 (1)	0	1 (<1)	3 (5)	3 (33)
7L+	3 (<1)	0	0	2 (3)	1 (11)
	**Initial BV Regimen for PTCL, *n* (%)**				
	**N = 221**	***n* = 154**	***n* = 56**	***n* = 9**	***n* = 2**
2L	133 (60)	133 (86)	-	-	-
3L	62 (28)	17 (11)	45 (80)	-	-
4L	16 (7.2)	2 (1.3)	8 (14)	6 (67)	-
5L	9 (4.1)	1 (0.6)	3 (5.4)	3 (33)	2 (100)
6L	1 (0.5)	1 (0.6)	0 (0)	0 (0)	0 (0)

**Abbreviations**: 2L, second line of therapy; 3L, third line of therapy; 4L, fourth line of therapy; 5L, fifth line of therapy; 6L, sixth line of therapy; 7L+, seventh and later lines of therapy; BV, brentuximab vedotin; BV-M, brentuximab vedotin monotherapy; cHL, classical Hodgkin lymphoma; PTCL, peripheral T-cell lymphoma.

**Table 3:** Subsequent treatment post-BV-M, -BV-C, or -non-BV retreatment at index.

Subsequent Treatment ^a^	cHL, Index Treatment			PTCL, Index Treatment		
	BV-M *n* = 564	BV-C *n* = 276	Non-BV *n* = 900	BV-M *n* = 221	BV-C *n* = 73	Non-BV *n* = 336
Systemic therapy, *n* (%)	174 (31)	108 (39)	300 (33)	89 (40)	40 (55)	134 (40)
BV-M	57 (10)	15 (5)	0 (0)	40 (18)	4 (6)	0 (0)
BV-C	8 (1)	29 (11)	0 (0)	3 (1)	9 (12)	0 (0)
Non-BV	109 (19)	64 (23)	300 (33)	46 (21)	27 (37)	134 (40)
Time to subsequent systemic therapy, median (IQR), mo	7 (4–12)	6 (3–9)	6 (3–13)	8 (5–13)	7 (4–11)	4 (2–6)
SCT, *n* (%)	3 (0.5)	7 (2.5)	54 (6.0)	6 (2.7)	1 (1.4)	9 (2.7)
Autologous	0 (0)	3 (1.1)	40 (4.4)	2 (0.9)	0 (0)	4 (1.2)
Allogeneic	3 (0.5)	4 (1.4)	14 (1.6)	4 (1.8)	1 (1.4)	5 (1.5)
Time to subsequent SCT, median (IQR), mo	5 (4–9)	3 (3–6)	3 (1–4)	4 (2–5)	5	3 (0–4)

^a^, Subsequent treatment received post-index therapy. **Abbreviations:** BV, brentuximab vedotin; BV-C, brentuximab vedotin combination therapy; BV-M, brentuximab vedotin monotherapy; cHL, classical Hodgkin lymphoma; IQR, interquartile range; mo, months; PTCL, peripheral T-cell lymphoma; SCT, stem cell transplantation.

## Data Availability

The data underlying this article will be shared on reasonable request to the corresponding author.
